# Genetic Algorithm and Greedy Strategy-Based Multi-Mission-Point Route Planning for Heavy-Duty Semi-Rigid Airship

**DOI:** 10.3390/s22134954

**Published:** 2022-06-30

**Authors:** Shaoxing Hu, Bingke Wang, Aiwu Zhang, Yiming Deng

**Affiliations:** 1 School of Mechanical Engineering and Automation, Beihang University, Beijing 100191, China; 1920wangbk@buaa.edu.cn; 2 Key Laboratory of 3D Information Acquisition and Application, Ministry of Education, Capital Normal University, Beijing 100048, China; 3 Center for Geographic Environment Research and Education, Capital Normal University, Beijing 100048, China; 4 Nondestructive Evaluation Laboratory, Department of Electrical and Computer Engineering of the College of Engineering, Michigan State University, East Lansing, MI 48824, USA; dengyimi@egr.msu.edu

**Keywords:** multi-mission-point, route planning, minimum turning radius, optimal flight sequence, shortest route

## Abstract

The large volume and windward area of the heavy-duty semi-rigid airship (HSA) result in a large turning radius when the HSA passes through every mission point. In this study, a multi-mission-point route planning method for HSA based on the genetic algorithm and greedy strategy is proposed to direct the HSA maneuver through every mission point along the optimal route. Firstly, according to the minimum flight speed and the maximum turning slope angle of the HSA during turning, the minimum turning radius of the HSA near each mission point is determined. Secondly, the genetic algorithm is used to determine the optimal flight sequence of the HSA from the take-off point through all the mission points to the landing point. Thirdly, based on the optimal flight sequence, the shortest route between every two adjacent mission points is obtained by using the route planning method based on the greedy strategy. By determining the optimal flight sequence and the shortest route, the optimal route for the HSA to pass through all mission points can be obtained. The experimental results show that the method proposed in this study can generate the optimal route with various conditions of the mission points using simulation studies. This method reduces the total voyage distance of the optimal route by 18.60% on average and improves the flight efficiency of the HSA.

## 1. Introduction

The heavy-duty semi-rigid airship (HSA) is different from ordinary aerial photography unmanned airships as the HSA has a large volume and can carry a heavy load for a long voyage [1,2]. As a result, many HSAs have been widely used to perform observation, transportation, and other missions across multiple cities or regions [3,4,5], such as Germany Zeppelin NT airship [6], American ML866 airship [7], and China ASQ-HAA380 [8], to name a few. The HSAs are also different from airplanes due to their large volume and windward area, which result in a larger turning radius when performing flight missions. Therefore, in the HSA route planning, the shortest route between two mission points is not a straight line but a curve. How to obtain the optimal route so that HSAs can accurately pass through all mission points, e.g., cities or regions, in sequence with the shortest flight route identified to improve flight efficiency is critically important, however, multi-mission-point route planning remains challenging.

The multi-mission-point route planning problem of the HSA can actually be reduced to a traveling salesman problem (TSP) to solve [9], which is a famous combinatorial optimization problem. For HSA route planning, the problem can be specifically defined as finding the shortest loop that does not repeatedly pass through all target cities [10,11,12]. To the best knowledge of the authors, this problem has been extensively studied with numerous algorithms developed for solving TSP problems including the greedy algorithm; genetic algorithm [13,14,15]; simulated annealing algorithm [16,17]; ant colony algorithm [18,19]; and particle swarm optimization algorithm [20,21], etc. Lideng, P. and Xiaofeng, H. [22] proposed a simple heuristic greedy method to solve the traveling salesman problem by using the distance information between cities. Dengwu, M. and Wen, Y. [14] proposed an optimization method based on an adaptive pseudo-parallel genetic algorithm based on a basic genetic algorithm. The optimal flight route of the aircraft obtained by this method strictly passes through the starting point and the target point of the aircraft. X. Yue, W. Zhang [23] proposes a UAV route planning method based on the K-means algorithm and simulated annealing algorithm. The K-means algorithm is used to classify the target points, and the simulated annealing algorithm is used to draw the shortest route for all the target points that the UAV will take, and maximize the UAV cruise coverage. The algorithm improves the overall efficiency and cruise coverage of the UAV. X. Chen, Y. Dai [24] proposed an ant colony algorithm integrating the genetic algorithm to solve the path planning problem. The algorithm combines the advantages of ant colony algorithm and genetic algorithm and reduces the number of iterations of the optimal solution as well as the calculation time and cost. Based on the particle swarm optimization algorithm, Shu-Juan, T. and Ke, Z. [25] which improved the global search ability of route planning and obtained a more authoritative optimal route, this method solves the problem of single-machine path planning problem detection and multi-machine cooperation, and achieves good results. Although various algorithms have been proposed in the above literatures to solve the TSP in aircraft route planning through optimization algorithms or multi-algorithm fusion with success to some extent, it is worth noting that the influence of aircraft large turning radius on route planning was never considered.

Li, R. and Xu, H. [26] proposed a UAV path planning approach based on modified ant colony algorithm and DUBINS curve. The DUBINS curve is used to smooth the turning angle to obtain a shorter and smoother flight path and improve the operation efficiency of the UAV. Cheng, J. and Hu, X. [27] proposed an improved ant colony algorithm that can solve feasible paths and speed up the convergence speed. At the same time, the DUBINS curve is used to curve the solution path, so that the solution path can meet the requirements of the UAV flight curvature. Hansen, K.D. and Cour-Harbo, A.L. [28] proposed a variable radius trajectory generation and waypoint planning method based on DUBINS curve. This method proposes an improved genetic algorithm, which optimizes the continuous heading and target speed of the waypoints while optimizing the combined sequence. At the same time, the generation method of DUBINS curve with variable radius is introduced. The studies from the above literature use the DUBINS curve to solve the influence of the turning radius on the route when planning the route.

The DUBINS curve is the shortest path connecting two points under the constraints of curvature and tangent direction at the specified start and end points, and the target can only travel forward. Under the constraints, there will be multiple curves feasible, so the set of DUBINS curve D = {LSL, RSR, RSL, LSR, RLR, LRL}. The shortest route in the DUBINS curve set is the optimal solution [29,30]. However, the DUBINS curve has limitations, limiting the direction of the target and the direction of speed at the beginning and ending. Therefore, the optimal solution in the DUBINS curve set is only the optimal solution in the specific beginning and ending speed directions.

Table 1 summarizes the influence of whether the turning radius of the route planning algorithm proposed in the above studies was considered in route planning.

**Figure 1:**
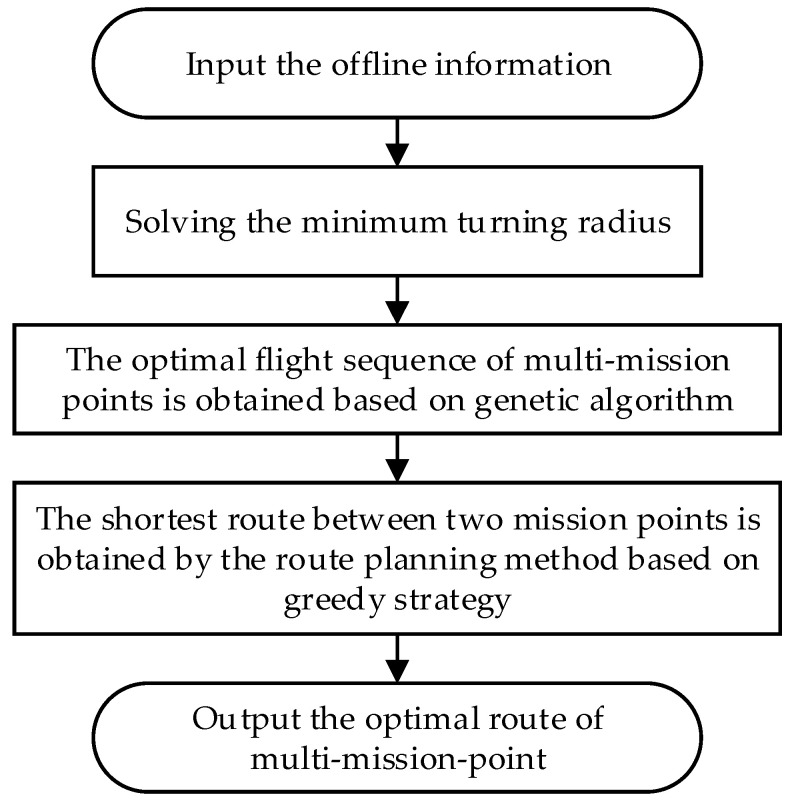
The overall flow chart of the route planning algorithm proposed. The input offline information includes HSA take-off and landing points and route mission points, the minimum flight speed, and the maximum turning slope angle of the HSA during turning.

When planning the route of the HSA, using the naive global search algorithm solely to find the optimal route will increase the computational time significantly and make it infeasible. Therefore, to address the aforementioned challenges, a multi-mission-point route planning method for HSA based on the genetic algorithm and greedy strategy is proposed in this paper. Firstly, according to the minimum flight speed and the maximum turning slope angle of the HSA, the minimum turning radius of the HSA near each mission point is determined. Secondly, the global search ability of the genetic algorithm is used to determine the optimal flight sequence of the HSA from the take-off point through all the mission points back to the landing point. Thirdly, based on the optimal flight sequence, a route planning method based on the greedy strategy is proposed, which uses the greedy strategy to decompose the optimal route problem of all mission points into the local optimal route problem of every two adjacent mission points. Then, this method traverses the multi-mission points in the optimal flight sequence in turn to obtain the shortest route between every two adjacent mission points. Finally, the optimal route of the HSA from the take-off point, through all the mission points, and back to the landing point is obtained. Figure 1 shows the flow chart of the proposed method that gives a holistic overview of the route planning algorithm.

The method proposed in this paper combines the global search ability of the genetic algorithm and the local optimal characteristics of the greedy strategy, which reduces the amount of calculation and improves the computing efficiency. At the same time, this method can accurately generate the optimal route and improve the flight efficiency. It can be seen from Table 1 that the route planning algorithms proposed in the literature [14,22,23,24,25] do not consider the influence of the turning radius, and the literature [26,27,28] proposed to use the DUBINS curve to solve the influence of the turning radius on the route. The DUBINS curve, however, has limitations, limiting the direction in which the target is heading and the speed direction at the beginning and ending. Therefore, the method proposed in this paper is compared with the multi-mission-point route planning method based on the DUBINS curve, and the results show that the total voyage of the optimal route obtained by the proposed method is reduced by 18.60% on average.

The rest of the paper is organized as follows: Section 2 introduces the multi-mission-point route planning method for the HSA. In Section 3, the experimental analysis is carried out. In Section 4, the findings are discussed. Finally, in Section 5, the conclusions are drawn.

## 2. Materials and Methods

In this section, the minimum turning radius of HSA is first introduced, and then the two stages of the method are introduced in detail. First, the optimal flight sequence of the multi-mission points is obtained based on the genetic algorithm. Second, the route planning method based on the greedy strategy obtains the shortest route between every two adjacent mission points in the optimal flight sequence.

### 2.1. Minimum Turning Radius

Since the HSA has a large volume and windward area, there is a large turning radius when passing through each mission point. Therefore, in the HSA route planning, the shortest route between two mission points is not a straight line but a curve. It is necessary to consider the influence of the turning radius on route planning. The turning radius of an airship [31,32] can be found by:

(1) $$R=\frac{TAS^{2}}{g\times \tan\gamma }$$

(2) $$TAS=IAS\sqrt{\frac{Pa}{Pa_{0}}}$$

where *TAS* is the vacuum velocity of the airship; *IAS* is the indicated airspeed of the airship; *g* is the local gravitational acceleration; γ is the slope angle when the airship turns; Pa is the current external air pressure value of the flight; $Pa_{0}$ is the standard sea level pressure value.

From Equation (1), it can see that the turning radius of the HSA is affected by the flight speed and the turning slope angle. The HSA has a fixed maximum turning slope angle during turning, so the smaller the flight speed of the HSA is during turning, the smaller the turning radius will be. Before route planning, according to the minimum flight speed $V_{\min}$ of the HSA and the maximum turning slope angle $\gamma_{\max}$ during turning, the minimum turning radius R of the HSA during turning can be obtained by Equations (1) and (2). Therefore, in the following route planning, the turning radius of the HSA passing through each mission point adopts the minimum turning radius R.

### 2.2. Genetic Algorithm Based Approach for Optimal Flight Sequence of Mission Points

After the HSA enters the working altitude, it will traverse all the selected mission points from the take-off point. References [9,33] simplified this problem as a traveling salesman problem, which aims to find a closed loop with the shortest distance in a series of nodes. As every route of the HSA starts from a fixed take-off point, the route planning problem for HSA can be described as a closed loop that traverses *n* mission points from the take-off point. In this way, the route distance of the HSA can be shortened, and the flight efficiency can be improved as well.

Among the various algorithms for solving the TSP problem, the genetic algorithm is an intelligent algorithm that searches for the optimal solution by simulating the natural evolution process. Furthermore, the genetic algorithm has the global search ability, which has a good effect on the TSP problem to quickly obtain better optimization results. It is worth noting from the above analysis that the influence of turning radius needs to be considered when HSA turns. In order to improve the flight efficiency of an airship, HSA adopts the minimum turning radius R. Therefore, before route planning, this paper investigated and demonstrated the global search ability of the genetic algorithm to obtain the optimal flight sequence of HSA passing through the multi-mission points. The specific steps of the proposed genetic algorithm are as follows:

1. Input the coordinates of take-off and landing point $P_{O}$ and mission points $P_{i}(i=1,2,3,\cdots ,n)$, and the number of nodes N=n+1;
2. Set the maximum genetic algebra, crossover probability, and mutation probability;
3. Calculate the distance $D_{ij}(i,j=1,2,3,\cdots ,N)$ between every two adjacent mission points;
4. Initialize the population and randomly generate multiple individuals starting from the take-off point;
5. Selection operation: calculate the fitness value of each individual in the population, and select the individual with a large fitness value as the new population to replace the original population;
6. Crossover operation: every two adjacent individuals in the population have a certain crossover probability. According to the single point crossover, the partial node sequences of the same length in the two adjacent individuals are cross exchanged to generate new individuals to replace the original individuals;
7. Mutation operation: each individual in the population has a certain mutation probability. The individual performs partial mutation, that is, two nodes in the node sequence are randomly selected and their sequence is exchanged;
8. Repeat steps 5–7 until the genetic algebra is the maximum genetic algebra, and output the individual with the maximum fitness in the evolutionary process;
9. According to the node sequence in the optimal solution individual, the coordinates of take-off and landing point, and mission points are output.

Note: the fitness value is the reciprocal of the total distance of the route.

### 2.3. Route Planning Method Based on the Greedy Strategy

The greedy strategy means that the agent always makes the best choice at present, in other words, the choice made by the greedy strategy is only a locally optimal solution in a certain sense. The main idea of the greedy strategy is to divide the problem into several sub-problems, solve each sub-problem to obtain the local optimal solution, and finally synthesize the local optimal solutions of all sub-problems into the optimal solution for the original problem.

From the above, the minimum turning radius during the HSA flight and the optimal flight sequence of the HSA passing through all multi-mission points can be obtained. Thus, the main goal of route planning becomes how to find an optimal route with the shortest distance and improve flight efficiency. Therefore, this paper proposes a route planning method based on the greedy strategy. This method adopts the idea of the greedy strategy to decompose the optimal route problem of all mission points into a local optimal route problem of every two adjacent mission points. The novel method reduces the computational complexity and improves the computational efficiency. Additionally, this method helps to traverse the multi-mission points in the optimal flight sequence in turn to determine the shortest route between every two adjacent mission points. Finally, the complete optimal route of the HSA from the take-off point to the landing point through all mission points can be obtained.

In order to simplify the problem and facilitate efficient calculation, this paper does not consider the direction of HSA at take-off and landing points, the following assumptions are proposed and met:

**Assumption** **1.** *The HSA takes off along the positive semi-axis direction of the* X *axis of the global coordinate system of the input multi-mission points.*

**Assumption** **2.** *The HSA can return to the landing point from any direction.*

**Assumption** **3.** *The take-off and landing point of the HSA coincide with the same point.*

The detailed steps of the proposed route planning method are presented as follows:

1. Input the initial information, such as the coordinates of the multi-mission points of the optimal flight sequence of the HSA;
2. Translation transformation of the coordinate system, the translation of the global coordinate system of the input multi-mission points is transformed into the local coordinate system of the mission point $P_{i+1}$, and the parameter expression of the coordinates of the circle center $O_{i+1}$ can be obtained;
3. Determine the coordinates of the circle center $O_{i}{}^{\prime }$ in the route $P_{i}P_{i+1}$, according to the coordinates of the circle center $O_{i}$ in the route $P_{i-1}P_{i}$, the coordinates of the circle center $O_{i}{}^{\prime }$ can be determined;
4. Determine the shortest route of the route $P_{i}P_{i+1}$, the expression of the total voyage distance of the route $P_{i}P_{i+1}$ is expressed by the coordinates of the circle center $O_{i+1}$, and the shortest route of the route $P_{i}P_{i+1}$ is determined by changing the coordinates of the circle center $O_{i+1}$;
5. Coordinate transformation of the shortest route, converting the shortest route from the local coordinate system back to the global coordinate system;

Note: Steps 2–5 could be repeated if necessary. When traversing back to the landing point of the HSA, the method will output the optimal route of the HSA from the take-off point through all the mission points back to the landing point.

**Figure 2:**
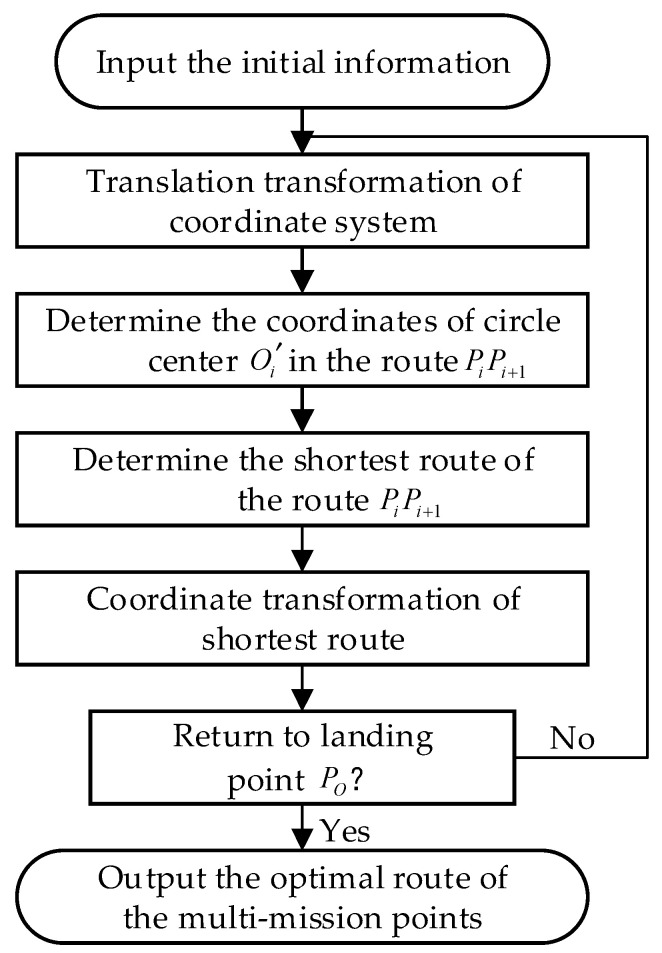
Flow chart of route planning method based on the greedy strategy. The initial input information includes the coordinates of the multi-mission points of the optimal flight sequence of the HAS and the coordinates of the circle center $O_{O}$ of the take-off point $P_{O}$.

The flow chart of this method is shown in Figure 2 with detailed algorithms described as follows:

#### 2.3.1. Input the Initial Information

From the above, the optimal flight sequence of the HSA from the take-off point, through all the mission points, and back to the landing point can be obtained by using the global search ability of the genetic algorithm. At the same time, according to Assumption 1, the HSA takes off along the positive semi-axis direction of the X axis of the global coordinate system OXY, so the coordinates of the circle center $O_{O}$ of the take-off point $P_{O}$ are set as $\left(x_{P_{O}},y_{P_{O}}+R\right)$.

Therefore, the input initial information includes the coordinates of the multi-mission points of the optimal flight sequence of the HSA and the coordinates $\left(x_{P_{O}},y_{P_{O}}+R\right)$ of the circle center $O_{O}$.

#### 2.3.2. Translation Transformation of the Coordinate System

The input multi-mission-point coordinates are located in the global coordinate system OXY, in order to obtain the shortest route $P_{i}P_{i+1}$ from the mission point $P_{i}$ to the mission point $P_{i+1}$ for convenience. The global coordinate system OXY is translated to the position where the origin coincides with the mission point $P_{i+1}$, so the local coordinate system $O^{\prime }X^{\prime }Y^{\prime }$ of the mission point $P_{i+1}$ is obtained. The translation transformation equation of the coordinate system is:

(3) $$\{\begin{array}{c} x_{i}{}^{\prime }=x_{i}-x_{P_{i+1}}\\ y_{i}{}^{\prime }=y_{i}-y_{P_{i+1}} \end{array}$$

where $x_{i}{}^{\prime },y_{i}{}^{\prime }$ are the x-coordinate and y-coordinate of each point in the local coordinate system $O^{\prime }X^{\prime }Y^{\prime }$; $x_{i},y_{i}$ are the x-coordinate and y-coordinate of each point in the global coordinate system OXY; $x_{P_{i+1}},y_{P_{i+1}}$ are the x-coordinate and y-coordinate of the mission point $P_{i+1}$ in the global coordinate system OXY.

The coordinates of the circle center $O_{i+1}$ of the mission point $P_{i+1}$ in local coordinate system $O^{\prime }X^{\prime }Y^{\prime }$ are parameterized as follows:

(4) $$\{\begin{array}{c} x_{O_{i+1}}{}^{\prime }=R\cos\theta \\ y_{O_{i+1}}{}^{\prime }=R\sin\theta \end{array},(\theta \in [0,2\pi ))$$

where $x_{O_{i+1}}{}^{\prime },y_{O_{i+1}}{}^{\prime }$ are the x-coordinate and y-coordinate of the circle center $O_{i+1}$ in the local coordinate system $O^{\prime }X^{\prime }Y^{\prime }$; θ∈[0,2π) is the angle parameter, where each parameter θ corresponds to a circle center $O_{i+1}$ coordinate. Under different circle center $O_{i+1}$ coordinates, the total voyage distance of the route $P_{i}P_{i+1}$ will be different. Therefore, by changing the value of θ, when the total voyage distance of the satisfied route is the smallest, the corresponding route will be the shortest route.

#### 2.3.3. Determine the Coordinates of the Circle Center $O_{i}{}^{\prime }$ in the Route $P_{i}P_{i+1}$

According to the shortest route $P_{i-1}P_{i}$ from the mission point $P_{i-1}$ to the mission point $P_{i}$, the coordinates of the circle center $O_{i}$ of the mission point $P_{i}$ can be obtained. Then the mission point $O_{i}$, the mission point $P_{i}$, and the mission point $P_{i+1}$ form a corner $∠O_{i}P_{i}P_{i+1}$. The size of $∠O_{i}P_{i}P_{i+1}$ can be divided into two cases: $∠O_{i}P_{i}P_{i+1}\leq 90{}^{\circ}$ and $∠O_{i}P_{i}P_{i+1}>90{}^{\circ}$, which are discussed separately as follows:

Case 1: $∠O_{i}P_{i}P_{i+1}\leq 90{}^{\circ}$:

When $∠O_{i}P_{i}P_{i+1}\leq 90{}^{\circ}$, the circle center $O_{i}{}^{\prime }$ of the mission point $P_{i}$ in the route $P_{i}P_{i+1}$ coincides with the circle center $O_{i}$ of the mission point $P_{i}$ in the route $P_{i-1}P_{i}$, then the coordinates of the circle center $O_{i}{}^{\prime }$ are:

(5) $$\{\begin{array}{c} x_{O_{i}{}^{\prime }}{}^{\prime }=x_{O_{i}}{}^{\prime }\\ y_{O_{i}{}^{\prime }}{}^{\prime }=y_{O_{i}}{}^{\prime } \end{array}$$

where $x_{O_{i}{}^{\prime }}{}^{\prime },y_{O_{i}{}^{\prime }}{}^{\prime }$ are the x-coordinate and y-coordinate of the circle center $O_{i}{}^{\prime }$ in the local coordinate system $O^{\prime }X^{\prime }Y^{\prime }$; $x_{O_{i}}{}^{\prime },y_{O_{i}}{}^{\prime }$ are the x-coordinate and y-coordinate of the circle center $O_{i}$ in the local coordinate system $O^{\prime }X^{\prime }Y^{\prime }$.

Case 2: $∠O_{i}P_{i}P_{i+1}>90{}^{\circ}$:

When $∠O_{i}P_{i}P_{i+1}>90{}^{\circ}$, the circle center $O_{i}{}^{\prime }$ of the mission point $P_{i}$ in the route $P_{i}P_{i+1}$ and the circle center $O_{i}$ of the mission point $P_{i}$ in route $P_{i-1}P_{i}$ are symmetrical about the mission point $P_{i}$, then the coordinates of the circle center $O_{i}{}^{\prime }$ are:

(6) $$\{\begin{array}{c} x_{O_{i}{}^{\prime }}{}^{\prime }=2\times x_{P_{i}}{}^{\prime }-x_{O_{i}}{}^{\prime }\\ y_{O_{i}{}^{\prime }}{}^{\prime }=2\times y_{P_{i}}{}^{\prime }-y_{O_{i}}{}^{\prime } \end{array}$$

where $x_{O_{i}{}^{\prime }}{}^{\prime },y_{O_{i}{}^{\prime }}{}^{\prime }$ are the x-coordinate and y-coordinate of the mission point $P_{i}$ in the local coordinate system $O^{\prime }X^{\prime }Y^{\prime }$.

#### 2.3.4. Determine the Shortest Route of the Route $P_{i}P_{i+1}$

The distance $D_{O_{i}{}^{\prime }P_{i+1}}$ from the circle center $O_{i}{}^{\prime }$ to the mission point $P_{i+1}$ is:

(7) $$D_{O_{i}{}^{\prime }P_{i+1}}=\sqrt{{\left(x_{O_{i}{}^{\prime }}{}^{\prime }-x_{P_{i+1}}{}^{\prime }\right)}^{2}+{\left(y_{O_{i}{}^{\prime }}{}^{\prime }-y_{P_{i+1}}{}^{\prime }\right)}^{2}}$$

where $x_{P_{i+1}}{}^{\prime },y_{P_{i+1}}{}^{\prime }$ are the x-coordinate and y-coordinate of the mission point $P_{i+1}$ in local coordinate system $O^{\prime }X^{\prime }Y^{\prime }$.

**Figure 3:**
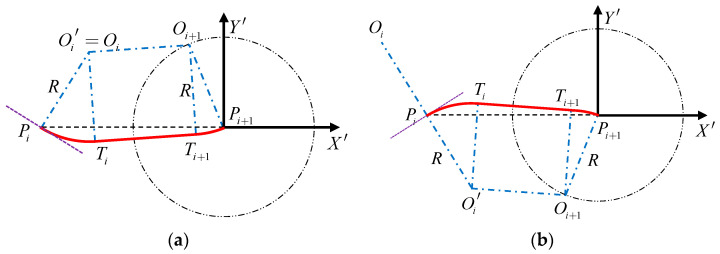
Schematic diagram of route planning when the distance $D_{O_{i}{}^{\prime }P_{i+1}}$ is less than the minimum turning radius R. (**a**) is the schematic diagram of route planning at $∠O_{i}P_{i}P_{i+1}\leq 90$, (**b**) is the schematic diagram of a route planning at $∠O_{i}P_{i}P_{i+1}>90$, the red line is the calculated shortest route.

Regarding the geometric relationship between the distance $D_{O_{i}{}^{\prime }P_{i+1}}$ and the minimum turning radius R, there are three cases: $D_{O_{i}{}^{\prime }P_{i+1}}>R$, $D_{O_{i}{}^{\prime }P_{i+1}}<R$, and $D_{O_{i}{}^{\prime }P_{i+1}}=R$, which are discussed separately as follows:

Case 1: $D_{O_{i}{}^{\prime }P_{i+1}}>R$:

As shown in Figure 3, when the distance $D_{O_{i}{}^{\prime }P_{i+1}}$ is greater than the minimum turning radius R, the route $P_{i}P_{i+1}$ includes three parts: the arc route where the mission point $P_{i}$ is located, the arc route where the mission point $P_{i+1}$ is located, and the straight line in the middle. Among them, the arc route where the mission point $P_{i}$ is located and the arc route where the mission point $P_{i+1}$ is located are both inferior arcs.

By defining the tangent point between the arc route where the mission point $P_{i}$ is located and the straight route as $T_{i}$; and defining the tangent point between the arc route where the mission point $P_{i+1}$ is located and the straight route as $T_{i+1}$, it can be seen from Figure 3 that the geometric relationship $∠P_{i}O_{i}{}^{\prime }O_{i+1}=∠P_{i}O_{i}{}^{\prime }T_{i}+90{}^{\circ}$ is valid, so

(8) $$\cos{\left(∠P_{i}O_{i}{}^{\prime }T_{i}\right)}^{2}+\cos{\left(∠P_{i}O_{i}{}^{\prime }O_{i+1}\right)}^{2}=1$$

which leads to

(9) $${\left(\frac{O_{i}{}^{\prime }P_{i}{}^{2}+O_{i}{}^{\prime }T_{i}{}^{2}-T_{i}P_{i}{}^{2}}{2\times O_{i}{}^{\prime }P_{i}\times O_{i}{}^{\prime }T_{i}}\right)}^{2}+{\left(\frac{O_{i}{}^{\prime }P_{i}{}^{2}+O_{i}{}^{\prime }O_{i+1}{}^{2}-O_{i+1}P_{i}{}^{2}}{2\times O_{i}{}^{\prime }P_{i}\times O_{i}{}^{\prime }O_{i+1}}\right)}^{2}=1$$

where $O_{i}{}^{\prime }P_{i}$ and $O_{i}{}^{\prime }T_{i}$ are equal to the minimum turning radius R; $T_{i}P_{i}$ is the distance from the tangent point $T_{i}$ to the mission point $P_{i}$; $O_{i}{}^{\prime }O_{i+1}$ is the distance from the circle center $O_{i+1}$ to the circle center $O_{i}{}^{’}$; $O_{i+1}P_{i}$ is the distance from the circle center $O_{i+1}$ to the mission point $P_{i}$.

The tangent point $T_{i}$ is located on the circle with center $O_{i}{}^{\prime }$, so $O_{i}{}^{\prime }T_{i}=R$, that is:

(10) $${\left(x_{O_{i}{}^{\prime }}{}^{\prime }-x_{T_{i}}{}^{\prime }\right)}^{2}+{\left(y_{O_{i}{}^{\prime }}{}^{\prime }-y_{T_{i}}{}^{\prime }\right)}^{2}=R^{2}$$

As it can be seen from the above, $∠O_{i}{}^{\prime }P_{i}P_{i+1}\leq 90{}^{\circ}$, and $∠O_{i}{}^{\prime }P_{i}P_{i+1}+∠T_{i}P_{i}P_{i+1}\leq 90{}^{\circ}$, so $∠T_{i}P_{i}P_{i+1}\leq 90{}^{\circ}$, that is:

(11) $$\frac{T_{i}P_{i}{}^{2}+P_{i}P_{i+1}{}^{2}-T_{i}P_{i+1}{}^{2}}{2T_{i}P\times P_{i}P_{i+1}}\geq 0$$

where $P_{i}P_{i+1}$ is the distance from the mission point $P_{i}$ to the mission point $P_{i+1}$; $T_{i}P_{i+1}$ is the distance from the tangent point $T_{i}$ to the mission point $P_{i+1}$.

It can be seen from Equations (9)–(11) that the coordinate $\left(x_{T_{i}}{}^{\prime },y_{T_{i}}{}^{\prime }\right)$ of the tangent point $T_{i}$ can be obtained. Similarly, the coordinate $\left(x_{T_{i+1}}{}^{\prime },y_{T_{i+1}}{}^{\prime }\right)$ of the tangent point $T_{i+1}$ can also be obtained.

Therefore, the route $P_{i}P_{i+1}$ includes arc route $P_{i}T_{i}$, straight route $T_{i}T_{i+1}$ and arc route $T_{i+1}P_{i+1}$. The voyage distance of each part of the route is:

(12) $$\left\{ \begin{array}{l} l_{1}=2R\times a\sin(\frac{\sqrt{{\left(x_{P_{i}}{}^{\prime }-x_{T_{i}}{}^{\prime }\right)}^{2}+{\left(y_{P_{i}}{}^{\prime }-y_{T_{i}}{}^{\prime }\right)}^{2}}}{2R})\\ l_{2}=\sqrt{{\left(x_{T_{i}}{}^{\prime }-x_{T_{i+1}}{}^{\prime }\right)}^{2}+{\left(y_{T_{i}}{}^{\prime }-y_{T_{i+1}}{}^{\prime }\right)}^{2}}\\ l_{3}=2R\times a\sin(\frac{\sqrt{{\left(x_{P_{i+1}}{}^{\prime }-x_{T_{i+1}}{}^{\prime }\right)}^{2}+{\left(y_{P_{i+1}}{}^{\prime }-y_{T_{i+1}}{}^{\prime }\right)}^{2}}}{2R}) \end{array}\right.$$

where $l_{1}$ is the distance of the arc route $P_{i}T_{i}$; $l_{2}$ is the distance of the straight route $T_{i}T_{i+1}$; $l_{3}$ is the distance of the arc route $T_{i+1}P_{i+1}$.

Then, the total voyage distance l of the route $P_{i}P_{i+1}$ is:

(13) $$l=l_{1}+l_{2}+l_{3}$$

Change the value of parameter θ(0≤θ<2π). When θ satisfies that the total voyage distance l of the route $P_{i}P_{i+1}$ at the minimum value, the corresponding route is the optimal route. At this time, the coordinates of the circle center $O_{i+1}$ corresponding to the θ are:

(14) $$\{\begin{array}{c} x_{O_{i+1}}{}^{\prime }=R\cos\theta \\ y_{O_{i+1}}{}^{\prime }=R\sin\theta \end{array}$$

**Figure 4:**
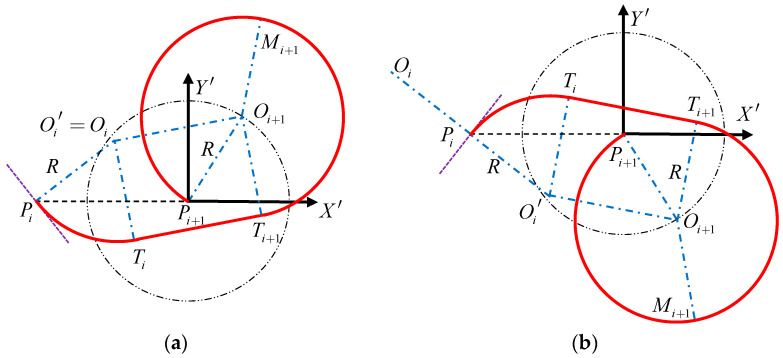
Schematic diagram of route planning when the distance $D_{O_{i}{}^{\prime }P_{i+1}}$ is less than the minimum turning radius R. (**a**) is the schematic diagram of route planning at $∠O_{i}P_{i}P_{i+1}\leq 90$, (**b**) is the schematic diagram of a route planning at $∠O_{i}P_{i}P_{i+1}>90$, the red line is the calculated shortest route.

Case 2: $D_{O_{i}{}^{\prime }P_{i+1}}<R$:

As shown in Figure 4, when the distance $D_{O_{i}{}^{\prime }P_{i+1}}$ is less than the minimum turning radius R, the route $P_{i}P_{i+1}$ includes three parts: the arc route where the mission point $P_{i}$ is located, the arc route where the mission point $P_{i+1}$ is located, and the straight line in the middle. Among them, the arc route where the mission point $P_{i}$ is located is the inferior arc, and the arc route where the mission point $P_{i+1}$ is located is the superior arc.

Similarly, let the tangent point between the arc route where the mission point $P_{i}$ is located and the straight route be $T_{i}$; let the tangent point between the arc route where the mission point $P_{i+1}$ is located and the straight route be $T_{i+1}$.

It can be seen from Equations (9)–(11) that the coordinate $\left(x_{T_{i}}{}^{\prime },y_{T_{i}}{}^{\prime }\right)$ of the tangent point $T_{i}$ and the coordinate $\left(x_{T_{i+1}}{}^{\prime },y_{T_{i+1}}{}^{\prime }\right)$ of the tangent point $T_{i+1}$ can be obtained.

Since the arc $T_{i+1}P_{i+1}$ is the superior arc, the arc $T_{i+1}P_{i+1}$ is equally divided into two sections, and the midpoint of the arc $T_{i+1}P_{i+1}$ is taken as $M_{i+1}$. According to the explicit method of locating the midpoint of the arc in the Cartesian plane mentioned in reference [34], the coordinates of the point $M_{i+1}$ of the arc $T_{i+1}P_{i+1}$ can be obtained as $\left(x_{M_{i+1}}{}^{\prime },y_{M_{i+1}}{}^{\prime }\right)$.

Therefore, the route $P_{i}P_{i+1}$ includes: arc route $P_{i}T_{i}$, straight route $T_{i}T_{i+1}$ arc route $T_{i+1}M_{i+1}$ and arc route $M_{i+1}P_{i+1}$. The voyage distance of each part of the route is:

(15) $$\left\{ \begin{array}{l} l_{1}=2R\times a\sin(\frac{\sqrt{{\left(x_{P_{i}}{}^{\prime }-x_{T_{i}}{}^{\prime }\right)}^{2}+{\left(y_{P_{i}}{}^{\prime }-y_{T_{i}}{}^{\prime }\right)}^{2}}}{2R})\\ l_{2}=\sqrt{{\left(x_{T_{i}}{}^{\prime }-x_{T_{i+1}}{}^{\prime }\right)}^{2}+{\left(y_{T_{i}}{}^{\prime }-y_{T_{i+1}}{}^{\prime }\right)}^{2}}\\ l_{4}=2R\times a\sin(\frac{\sqrt{{\left(x_{M_{i+1}}{}^{\prime }-x_{T_{i+1}}{}^{\prime }\right)}^{2}+{\left(y_{M_{i+1}}{}^{\prime }-y_{T_{i+1}}{}^{\prime }\right)}^{2}}}{2R})\\ l_{5}=2R\times a\sin(\frac{\sqrt{{\left(x_{M_{i+1}}{}^{\prime }-x_{P_{i+1}}{}^{\prime }\right)}^{2}+{\left(y_{M_{i+1}}{}^{\prime }-y_{P_{i+1}}{}^{\prime }\right)}^{2}}}{2R}) \end{array}\right.$$

where $l_{4}$ is the distance of the arc route $T_{i+1}M_{i+1}$; $l_{5}$ is the distance of the arc route $M_{i+1}P_{i+1}$.

Then, the total voyage distance l of the route $P_{i}P_{i+1}$ is:

(16) $$l=l_{1}+l_{2}+l_{4}+l_{5}$$

Similarly, change the value of parameter θ(0≤θ<2π). When θ satisfies that the total distance l of the route $P_{i}P_{i+1}$ at the minimum value, the corresponding route is the optimal route. At this time, the coordinates of the circle center $O_{i+1}$ corresponding to θ are shown in Equation (13).

**Figure 5:**
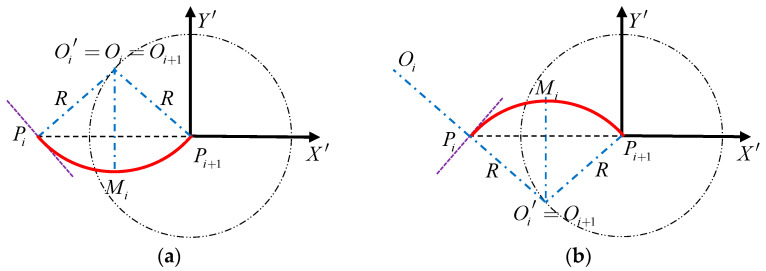
Schematic diagram of route planning when the distance $D_{O_{i}{}^{\prime }P_{i+1}}$ is equal to the minimum turning radius R. (**a**) is the schematic diagram of route planning at $∠O_{i}P_{i}P_{i+1}\leq 90$, (**b**) is the schematic diagram of a route planning at $∠O_{i}P_{i}P_{i+1}>90$, the red line is the calculated shortest route.

Case 3: $D_{O_{i}{}^{\prime }P_{i+1}}=R$:

As shown in Figure 5, when the distance $D_{O_{i}{}^{\prime }P_{i+1}}$ is equal to the minimum turning radius R, the mission point $P_{i}$ and the mission point $P_{i+1}$ are located on the arc of the circle center $O_{i}{}^{\prime }$ at the same time. At this time, the route $P_{i}P_{i+1}$ only includes the arc route $P_{i}P_{i+1}$.

In order to prevent the arc $P_{i}P_{i+1}$ from being the superior arc, the midpoint of the arc $P_{i}P_{i+1}$ is taken as $M_{i}$. Then according to the explicit method of locating the midpoint of the arc in the Cartesian plane mentioned in reference [34], the coordinates of the midpoint $M_{i}$ of the arc $P_{i}P_{i+1}$ can be obtained as $\left(x_{M_{i}}{}^{\prime },y_{M_{i}}{}^{\prime }\right)$.

Therefore, the route $P_{i}P_{i+1}$ includes arc route $P_{i}M_{i}$ and arc route $M_{i}P_{i+1}$. The voyage distance of each part of the route is:

(17) $$\{\begin{array}{c} l_{6}=2R\times \arcsin(\frac{\sqrt{{\left(x_{P_{i}}{}^{\prime }-x_{M_{i}}{}^{\prime }\right)}^{2}+{\left(y_{P_{i}}{}^{\prime }-y_{M_{i}}{}^{\prime }\right)}^{2}}}{2R})\\ l_{7}=2R\times \arcsin(\frac{\sqrt{{\left(x_{M_{i}}{}^{\prime }-x_{P_{i+1}}{}^{\prime }\right)}^{2}+{\left(y_{M_{i}}{}^{\prime }-y_{P_{i+1}}{}^{\prime }\right)}^{2}}}{2R}) \end{array}$$

where $l_{6}$ is the distance of the arc route $P_{i}M_{i}$; $l_{7}$ is the distance of the arc route $M_{i}P_{i+1}$.

Then, the total voyage distance l of the route $P_{i}P_{i+1}$ is:

(18) $$l=l_{6}+l_{7}$$

At this time, the circle center $O_{i+1}$ coincides with the circle center $O_{i}{}^{\prime }$, which coordinates are:

(19) $$\{\begin{array}{c} x_{O_{i+1}}{}^{\prime }=x_{O_{i}}{}^{\prime }\\ y_{O_{i+1}}{}^{\prime }=y_{O_{i}}{}^{\prime } \end{array}$$

#### 2.3.5. Coordinate Transformation of the Shortest Route

The shortest route $P_{i}P_{i+1}$ and the coordinates of the circle center $O_{i+1}$ in the above process are all located in the local coordinate system $O^{\prime }X^{\prime }Y^{\prime }$. In order to obtain the shortest route of the entire route, it necessary to convert back to the global coordinate system OXY. The translation transformation equation of the coordinate system is:

(20) $$\{\begin{array}{c} x_{i}=x_{i}{}^{\prime }+x_{P_{i+1}}\\ y_{i}=y_{i}{}^{\prime }+y_{P_{i+1}} \end{array}$$

Therefore, the shortest route $P_{i}P_{i+1}$ and the coordinates of the circle center $O_{i+1}$ in the global coordinate system OXY are obtained.

**Figure 6:**
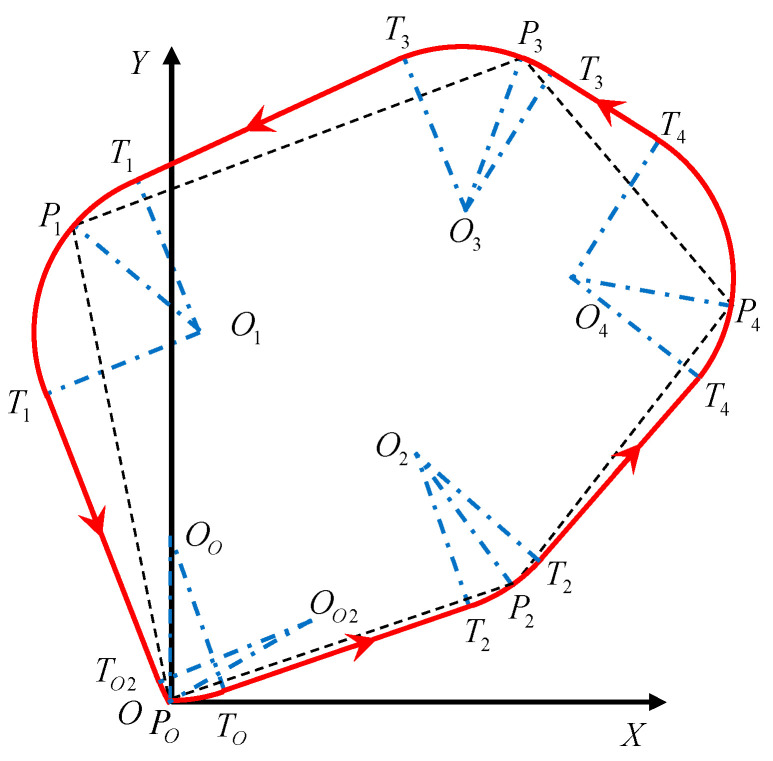
Schematic diagram of optimal route. $P_{O}$ is the take-off and landing point of the HSA, $P_{1},P_{2},P_{3},P_{4}$ are the input multi-mission points, $P_{O}\to P_{2}\to P_{4}\to P_{3}\to P_{1}\to P_{O}$ is the optimal flight sequence based on the genetic algorithm, and the red line is the optimal route obtained by the route planning method based on the greedy strategy.

By repeating steps 2–5 of the proposed route planning method based on the greedy strategy, the method traverses the mission points in the optimal flight sequence in turn to obtain the shortest route between every two adjacent mission points, and outputs the optimal route of the HSA as shown in Figure 6.

## 3. Experimental Analysis

**Figure 7:**
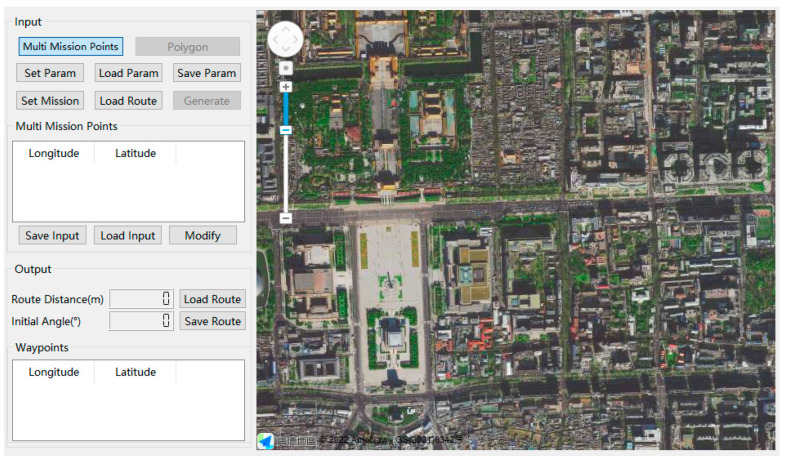
Layout of route planning simulation software developed for multi-mission-point route planning. After selecting the “Multi-Mission Points” mode, the user can click “Set Mission”, then click on the map to set the coordinates of the multi-mission points, and finally click “Generate”. And the optimal route will be displayed on the map.

To validate the method proposed in this paper, this paper leveraged the simulation software of the plant protection UAV route planning developed by the authors [35] and further developed simulation software for the multi-mission-point route planning of the HSA. The software interface and layout are shown in Figure 7.

**Figure 8:**
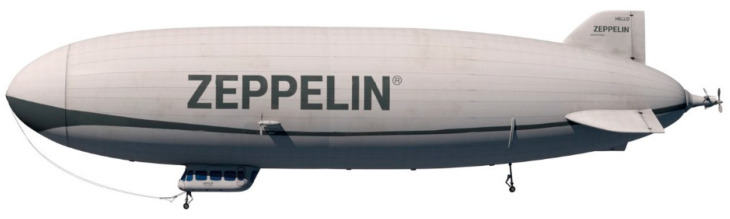
Representative HSA, Zeppelin NT airship adopted in the simulation study and validation.

**Figure 9:**
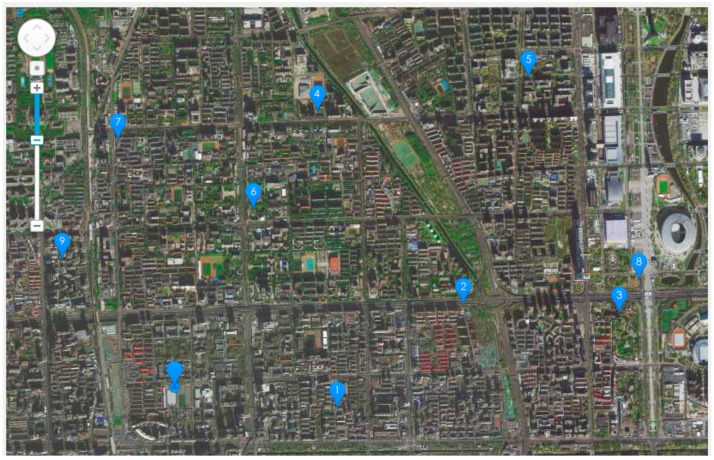
The mission point selection diagram, where the unnumbered point represents the take-off and landing point, and the marked points numbered 1~9 represent the nine mission points selected in sequence.

In this paper, the simulation study and analysis adopted the Zeppelin NT airship as a representative HSA as shown in Figure 8, which is 75 m long and 20 m wide. During the simulation, the flight speed of the HSA is set as $V_{\min}=100\;km/h$ and the maximum turning slope angle as $\gamma_{\max}=20{}^{\circ}$. Randomly selected multi-mission points are illustrated in Figure 9.

Firstly, according to the minimum flight speed $V_{\min}=100\;km/h$ and the maximum turning slope angle $\gamma_{\max}=20{}^{\circ}$ of the HSA when turning, by Equations (1) and (2), the minimum turning radius of the HSA can be obtained as:

(21) R=Vmin2g×tanγmax=1003.629.8×tan20°≈220 m

**Figure 10:**
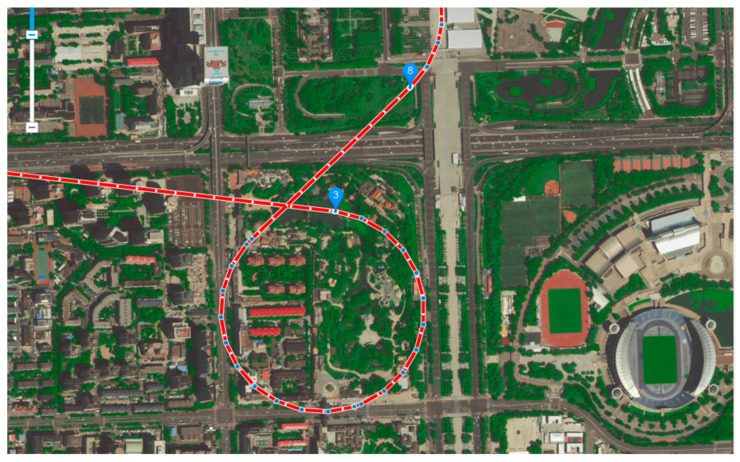
The partial enlarged view of the optimal route of mission points 8→3. The red line with arrows is the calculated best route, the marked points represent the mission point.

Secondly, the optimal flight sequence of the multi-mission points is obtained based on genetic algorithm. First, enter the take-off point coordinates of the HSA, and the coordinates of the randomly selected mission points 1~9. Then, set the maximum evolutionary generation to 1000, the crossover probability to 0.9, and the mutation probability to 0.1. Finally, based on the genetic algorithm, the optimal flight sequence of mission points 1~9 is obtained as: start→9→7→6→4→5→8→3→2→1→end;

Then, according to the route planning method based on the greedy strategy proposed in this paper, the multi-mission points in the optimal flight sequence are traversed in turn, and the shortest route between every two adjacent mission points start→9, 9→7, 7→6, 6→4, 4→5, 5→8, 8→3, 3→2, 2→1, and 1→end are obtained. Among them, the route of mission points 2→1 belongs to the situation where the distance $D_{O_{i}{}^{\prime }P_{i+1}}$ is greater than the minimum turning radius R in the above; the route of mission points 8→3 belongs to the situation where the distance $D_{O_{i}{}^{\prime }P_{i+1}}$ is less than the minimum turning radius R in the above. Figure 10 is the partial enlarged view of the shortest route of mission points 8→3.

**Figure 11:**
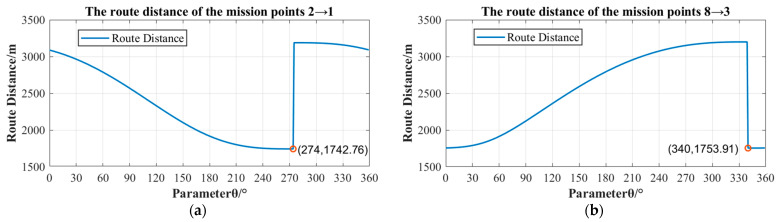
Schematic diagram of the route distance of two adjacent mission points corresponding to different parameters θ. (**a**) the relationship between parameter θ and the route distance of the mission points 2→1, (**b**) the relationship between parameter θ and the route distance of the mission points 8→3.

According to the route planning method based on the greedy strategy proposed in this paper, the shortest route of mission points 2→1 is solved. The specific steps are as follows:

First, the translation transformation of the coordinate system can obtain the parameter expression of the coordinates of the circle center $O_{1}$ of the mission point $P_{1}$. Second, according to the coordinates of the circle center $O_{2}$ of the mission point $P_{2}$ in the shortest route 3→2, the coordinates of the circle center $O_{2}{}^{\prime }$ of the mission point $P_{2}$ in the route 2→1 can be determined. Then, the distance $D_{21}$ from the mission point $P_{2}$ to the mission point $P_{1}$ is greater than the minimum turning radius R, so the expression of the total voyage distance of route 2→1 is as Equation (12). By changing the coordinates of the circle center $O_{1}$, the shortest route of the route can be determined. Finally, the relationship between the parameter θ and the route distance of the mission points 2→1 is shown in Figure 11a. When the parameter θ is 274°, the shortest distance of the route 2→1 is 1742.76 m. Similarly, the shortest route of mission points 8→3 is solved by the route planning method based on the greedy strategy proposed in this paper, and the relationship between the parameters θ and the route distance of the mission points 8→3 is shown in Figure 11b. When the parameter θ is 340°, the shortest distance of the route 8→3 is 1753.91 m.

**Figure 12:**
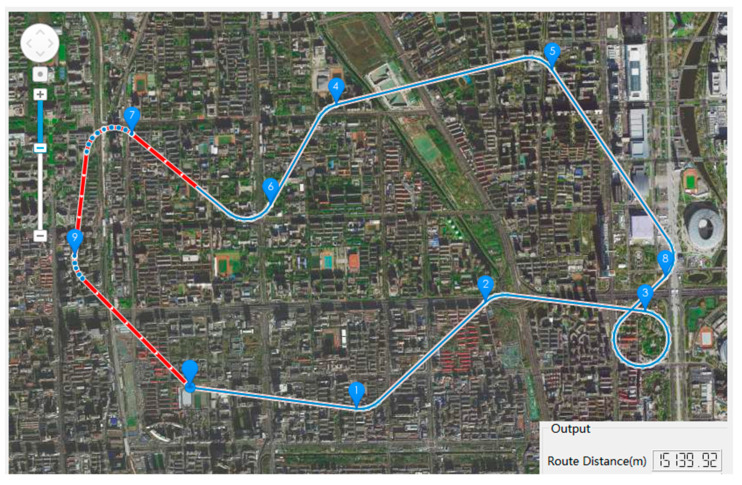
Schematic diagram of the output result of the route planning software. The blue line with arrows is the calculated best route, and the red line is the route that the simulated airship has traveled. And the output shows that the total route distance of the optimal route is 15,139.92 m.

Finally, the optimal route of the HSA from the take-off point, through all the mission points, and back to the landing point is obtained. The total voyage distance of the optimal route is 15,139.92 m, as shown in Figure 12.

**Figure 13:**
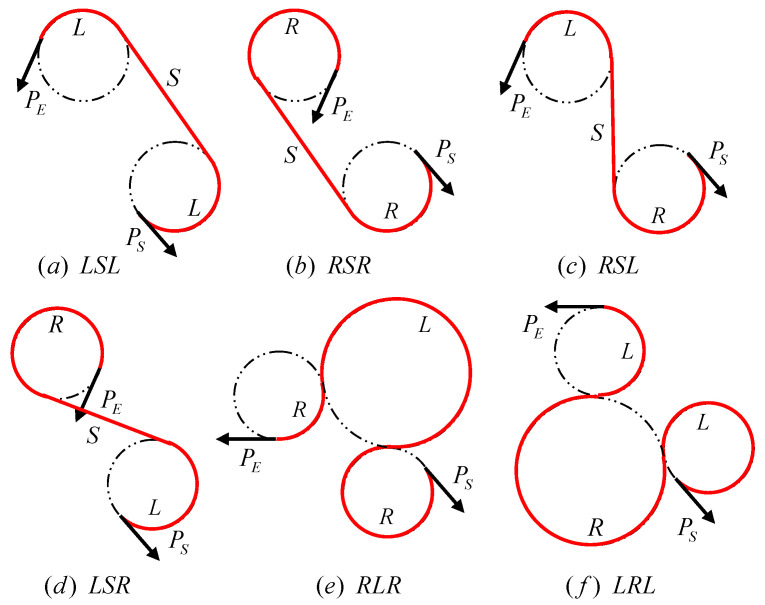
The main types of DUBINS path. $P_{S}$ represents the beginning speed direction, $P_{E}$ represents the ending speed direction,S represents the straight line, R represents the right turn, and L represents the left turn. (**a**) LSL means that the HSA starts from the beginning point $P_{S}$, first turns left, then goes straight line, and finally turns left to the ending point $P_{E}$. (**b**) RSR means that the HSA starts from the beginning point $P_{S}$, first turns right, then goes straight line, and finally turns right to the ending point $P_{E}$. (**c**) RSL means that the HSA starts from the beginning point $P_{S}$, first turns right, then goes straight line, and finally turns left to the ending point $P_{E}$. (**d**) LSR means that the HSA starts from the beginning point $P_{S}$, first turns left, then goes straight line, and finally turns right to the ending point $P_{E}$. (**e**) RLR means that the HSA starts from the beginning point $P_{S}$, first turns right, then turns left, and finally turns right to the ending point $P_{E}$. (**f**) LRL means that the HSA starts from the beginning point $P_{S}$, first turns left, then urns right, and finally turns left to the ending point $P_{E}$.

In the literature [26], the DUBINS curve is used to perform curve fitting on the solution path, and the influence of the turning radius on the route is solved, as shown in Table 1. The literature [29] proposed that the DUBINS curve has only six control combinations that describe all the shortest paths: LSL, RSR, RSL, LSR, RLR, and LRL, as shown in the Figure 13. Among them, the LSL control combination means that the HSA starts from the beginning point $P_{S}$, first turns left, then goes straight line, and finally turns left to reach the ending point $P_{E}$, as shown in Figure 13a.

**Figure 14:**
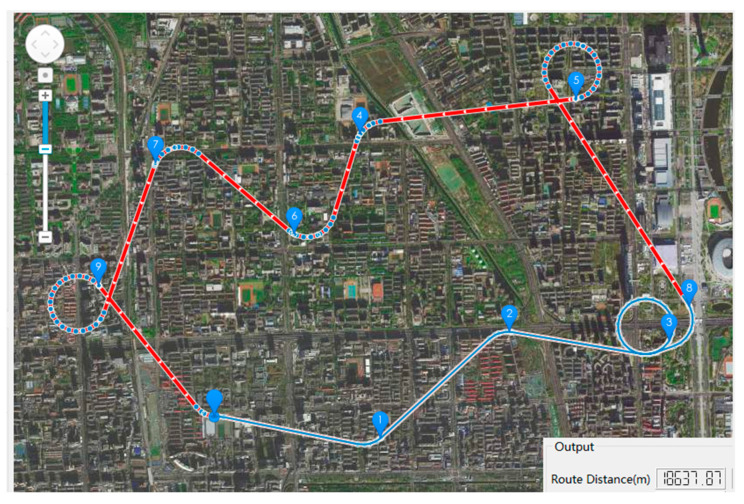
Schematic diagram of the simulation results of the multi-mission-point route planning method based on DUBINS curve. The red line with arrows is the calculated best route, and the blue line is the route that the simulated airship has traveled. And the output shows that the total route distance of the optimal route is 18,637.87 m.

The multi-mission-point route planning method based on DUBINS curve needs to determine the speed direction of the airship at the beginning and the ending when solving the shortest route between two mission points. Therefore, in the shortest route from the mission point $P_{i}$ to the mission point $P_{i+1}$, the speed direction of the beginning point $P_{i}$ can be determined by the shortest route from the mission point $P_{i-1}$ to the mission point $P_{i}$. The speed direction of the ending point $P_{i+1}$ points to the mission point $P_{i+2}$. The simulation results are shown in the Figure 14. Among them, the shortest route of the mission points 8→3 is the RSR in the six control combinations of the DUBINS curve. The shortest distance of route 8→3 is 1857.47 m. Compared with this method, the method proposed in this paper reduces the shortest distance by 5.58% in the route of mission points 8→3. At the same time, the total voyage distance of the optimal route is reduced by 18.77%, as shown in the route 1 in Table 2.

In order to prevent the accidental occurrence of the mission points selected for route 1, this paper randomly selects five sets of route data for comparative experiments. The take-off and landing points of routes 1~5 are the same, and the minimum turning radius of the airship is also R=220 m. The multi-mission points in the routes 1~5 are randomly selected, and the number of multi-mission points is shown in Table 2. The route planning method of multi-mission points based on DUBINS curve, and the multi-mission points route planning method proposed in this paper are used to plan the five sets of route data, respectively. The total voyages of the optimal routes of routes 1~5 are shown in Table 2. At the same time, the experimental results show that compared with the multi-mission-point route planning method based on DUBINS curve, the total voyage of the optimal routes of routes 1~5 obtained by the proposed method is reduced by 18.60% on average, as shown in Table 2.

From the simulation results, it can be seen that for various situations of multi-mission points, the multi-mission-point route planning method for HSA based on the genetic algorithm and greedy strategy proposed in this paper can generate the optimal route under the influence of turning radius. Furthermore, this method can ensure the accurate passing through multi-mission points and improve the flight efficiency of HSA. At the same time, the total voyage distance of the optimal route was reduced by 18.60% on average. The simulation results fully illustrate the feasibility of the route planning algorithm.

## 4. Discussion

Due to the large volume and large windward area of the HSA, there is a large turning radius when passing through each mission point. However, the route planning algorithms proposed in the literature [14,22,23,24,25] do not consider the influence of turning radius. The literature [26,27,28] proposed to use the DUBINS curve to solve the influence of the turning radius on the route, but the DUBINS curve has limitations, limiting the forward direction of the target and the speed direction at the beginning and ending. Therefore, the optimal solution in the DUBINS set is only the optimal solution in the specific beginning and ending speed directions. As such, this paper proposed a multi-mission-point route planning method for HSA based on genetic algorithm and greedy strategy. The method consists of the following two parts:

1. Optimal flight sequence: the global search ability of the genetic algorithm is used to determine the optimal flight sequence of the HSA from the take-off point through all the mission points back to the landing point.
2. Shortest flight route: a route planning method based on the greedy strategy is proposed, which uses the greedy strategy to decompose the optimal route problem of all mission points into the local optimal route problem of every two adjacent mission points. The shortest route between every two adjacent mission points can be obtained.

Through the optimal flight sequence and the shortest flight route, the optimal route of the HSA from the take-off point, through all the mission points, and back to the landing point is finally obtained. The advantages and characteristics of this method are as follows:

1. Under the influence of the large turning radius of the HSA, the optimal route can be generated quickly and accurately, and the flight efficiency of the HSA is greatly improved;
2. Combining the global search ability of genetic algorithm and the local optimal characteristics of the greedy strategy to improve overall computing efficiency;
3. This method has a broader range of applicability and can be applied to various complex mission situations between multiple mission points.

In the shortest flight route part, the method proposed in this paper can obtain the global shortest route between two adjacent mission points. The multi-mission-point route planning based on the DUBINS curve only obtains the local shortest route between two adjacent mission points in the case of a specific beginning and ending speed directions. Therefore, compared with the multi-mission-point route planning method based on DUBINS curve, the total voyage of the optimal route obtained by the proposed method is reduced by 18.60% on average.

However, there are still some remaining challenges and future work as this paper ignores problems such as the influence of environmental dynamics on the airship, especially the influence of wind, and the dynamic process of acceleration and deceleration and slope angle changes throughout the flight. The follow-up studies will take these into consideration. In addition to those identified limitations, this research lacks the evidence of flight experiments, which will be supplemented in the subsequent stage of research, where this proposed method is expected to be continuously improved with more adequate and realistic data.

## 5. Conclusions

In this study, a two-step approach considering the optimal flight sequence and the shortest route sequence is used to obtain the optimal route of the HSA from the take-off point, through all the mission points, and back to the landing point. The method proposed in this paper combines the advantages of the genetic algorithm and greedy strategy to enhance the adaptability of the algorithm and improve computational efficiency. The experimental results show that in view of the various conditions of the mission points, the method can generate the optimal route under the influence of turning radius, which greatly reduces the total voyage distance and improves the flight efficiency of the HSA. Compared with the multi-mission-point route planning method based on DUBINS curve, the total voyage of the optimal route obtained by the proposed method is reduced by 18.60% on average. At the same time, the feasibility and reliability of the route planning algorithm are demonstrated through experimental studies and simulation.

## Figures and Tables

**Table 1:** Various route planning algorithms.

Ref	Proposed Method	Consider Turning Radius Effects
[22]	A simple heuristic greedy method	NO
[14]	An optimization method based on an adaptive pseudo parallel genetic algorithm	NO
[23]	Combined the K-means clustering algorithm and simulated annealing algorithm	NO
[24]	An ant colony algorithm integrating genetic algorithm	NO
[25]	Combined particle swarm optimization algorithm and genetic algorithm	NO
[26]	A UAV path planning approach based on modified Ant colony algorithm and DUBINS curve	YES
[27]	Mixed ant colony Algorithm Based on DUBINS Path	YES
[28]	A variable radius trajectory generation and waypoint planning method based on DUBINS curve.	YES

**Table 2:** Comparison of simulation results of two route planning methods.

	The Number of Mission Points	Proposed Method	Based on DUBINS Curve	Optimization
Total voyage of route 1	9	15,139.92 m	18,637.87 m	18.77%
Total voyage of route 2	9	16,175.89 m	19,935.23 m	18.86%
Total voyage of route 3	9	13,350.61 m	16,446.26 m	18.82%
Total voyage of route 4	10	11,820.58 m	14,257.90 m	17.09%
Total voyage of route 5	24	42,841.27 m	53,194.56 m	19.46%
Average Optimization				18.60%

## Data Availability

Data sharing not applicable.

## References

[1] Manikandan M., Pant R.S. (2021). Research and advancements in hybrid airships—A review. Prog. Aerosp. Sci..

[2] Hu Y., Wu Z., Zhang D., Cui M. Random trajectory tracking for low altitude airship. Proceedings of the 2019 Chinese Control And Decision Conference (CCDC).

[3] Sarkar S., Gautam V. Low Carbon Airship. Proceedings of the AIAA Aviation 2019 Forum.

[4] Nayler A. Airship development world-wide—A 2001 review. Proceedings of the 1st AIAA, Aircraft, Technology Integration, and Operations Forum.

[5] Oebel A., Hofzumahaus A., Wahner A., Raak D., Broch S., Holland F., Rohrer F., Bohn B. In-situ measurements of vertical profiles of chemical tracers in the PBL using the airship Zeppelin NT. Proceedings of the ISARS 2010: International Symposium for the Advancement of Boundary Layer Remote Sensing.

[6] Watanabe H. Giant Rigid Airship and the Restoration Technique. Proceedings of the 18th AIAA Lighter-Than-Air Systems Technology Conference.

[7] Metlen T., Palazotto A.N., Cranston B. (2016). Economic optimization of cargo airships. CEAS Aeronaut. J..

[8] Hu S., Zhang A., Chai S. (2019). ASQ-MPHAAS: Multi-Payload Observation System From High Altitude Airship. IEEE Sens. J..

[9] Hu Z., Xia Q., Cai H. (2007). Random Searching Algorithms of Route Planning for High Altitude Airships. Comput. Simul..

[10] Wang Z., Duan H., Zhang X. An Improved Greedy Genetic Algorithm for Solving Travelling Salesman Problem. Proceedings of the 2009 Fifth International Conference on Natural Computation.

[11] Khalil M., Li J., Wang Y., Khan A. Algorithm to solve travel salesman problem efficently. Proceedings of the 2016 13th International Computer Conference on Wavelet Active Media Technology and Information Processing (ICCWAMTIP).

[12] Liu J., Li W. Greedy Permuting Method for Genetic Algorithm on Traveling Salesman Problem. Proceedings of the 2018 8th International Conference on Electronics Information and Emergency Communication (ICEIEC).

[13] Han Q., Cao W., Cui J. Research of Route Planning Based on Genetic Algorithm. Proceedings of the 2012 International Conference on Computer Science and Electronics Engineering.

[14] Ma D., Ye W., Lv X., Jiang W. Research on Airplane Route Planning Intelligent Decision System. Proceedings of the 2006 6th World Congress on Intelligent Control and Automation.

[15] Li L., Gu Q., Liu L. Research on Path Planning Algorithm for Multi-UAV Maritime Targets Search Based on Genetic Algorithm. Proceedings of the 2020 IEEE International Conference on Information Technology, Big Data and Artificial Intelligence (ICIBA).

[16] Ye G., Rui X. An improved simulated annealing andgenetic algorithm for TSP. Proceedings of the 2013 5th IEEE International Conference on Broadband Network & Multimedia Technology.

[17] Zheng X., Li Z., Li M. Optimized Shortest Path Model for Air Patrol of Shanghai Expo 2010 by Applying Simulated Annealing Algorithm. Proceedings of the 2011 Third Pacific-Asia Conference on Circuits, Communications and System (PACCS).

[18] Ismkhan H. (2017). Effective heuristics for ant colony optimization to handle large-scale problems. Swarm Evol. Comput..

[19] Chen X., Xu R., Zhao J. Multi-Objective Route Planning for UAV. Proceedings of the 2017 4th International Conference on Information Science and Control Engineering (ICISCE).

[20] Xin J., Zhong J., Li S., Sheng J., Cui Y. (2019). Greedy Mechanism Based Particle Swarm Optimization for Path Planning Problem of an Unmanned Surface Vehicle. Sensors.

[21] Zhong Y., Lin J., Wang L., Zhang H. (2018). Discrete comprehensive learning particle swarm optimization algorithm with Metropolis acceptance criterion for traveling salesman problem. Swarm Evol. Comput..

[22] Pan L., Huang X. (1998). A heuristic greedy method for the traveling salesman problem. J. Beijing Univ. Chem. Technol..

[23] Yue X., Zhang W. UAV Path Planning Based on K-Means Algorithm and Simulated Annealing Algorithm. Proceedings of the 2018 37th Chinese Control Conference (CCC).

[24] Chen X., Dai Y. Research on an Improved Ant Colony Algorithm Fusion with Genetic Algorithm for Route Planning. Proceedings of the 2020 IEEE 4th Information Technology, Networking, Electronic and Automation Control Conference (ITNEC).

[25] Tang S., Zhao K., Li D., Wang N. Route Planning Algorithm of Region Important Target Search Based on PSO. Proceedings of the 2018 IEEE 4th International Conference on Control Science and Systems Engineering (ICCSSE).

[26] Li R., Xu H., Dong J., Yu X. UAV path planning based on modified ant colony algorithm and DUBINS curves. Proceedings of the 2022 IEEE 6th Information Technology and Mechatronics Engineering Conference (ITOEC).

[27] Cheng J., Hu X., Xiao J., Zhang G., Zhou Q. Route planning of mixed ant colony algorithm based on Dubins path. Proceedings of the 2021 IEEE 16th Conference on Industrial Electronics and Applications (ICIEA).

[28] Hansen K.D., Cour-Harbo A.L. Waypoint planning with Dubins curves using genetic algorithms. Proceedings of the 2016 European Control Conference (ECC).

[29] Dubins L. (1957). On curves of Minimal lenght with a constraint on average curvature and with prescribed initial and terminal positions and tangents. Amer. J. Math..

[30] Song X., Hu S. 2D path planning with dubins-path-based A algorithm for a fixed-wing UAV. Proceedings of the 2017 3rd IEEE International Conference on Control Science and Systems Engineering (ICCSSE).

[31] Zhan J., Xie W., Guo Q., Zhang P. An Improved UAV Coverage Search Route Planning Method. Proceedings of the 2018 IEEE CSAA Guidance, Navigation and Control Conference (CGNCC).

[32] Li W., Hu Y., Sun S., Li J., Chu L. (2019). UAV turning path planning algorithm based on minimum turning radius. Comput. Eng. Des..

[33] Wu L., Li Z. (2011). The Research of Route Planning for Stratospheric Airships Based on Genetic Algorithms. Spacecr. Recovery Remote Sens..

[34] Akulov G.V., Akulov O.G. (2013). A Lesson Plan with an Arc Midpoint. J. Math. Sci. Collab. Explor..

[35] Hu S., Xu T., Wang B. (2021). Route-Planning Method for Plant Protection Rotor Drones in Convex Polygon Regions. Sensors.

